# Fabrication of a Robust In_2_O_3_ Nanolines FET Device as a Biosensor Platform

**DOI:** 10.3390/mi12060642

**Published:** 2021-05-31

**Authors:** Zetao Zhu, Takao Yasui, Quanli Liu, Kazuki Nagashima, Tsunaki Takahashi, Taisuke Shimada, Takeshi Yanagida, Yoshinobu Baba

**Affiliations:** 1 Department of Biomolecular Engineering, Graduate School of Engineering, Nagoya University, Nagoya 464-8603, Japan; liu.quanli@g.mbox.nagoya-u.ac.jp (Q.L.); shimada@chembio.nagoya-u.ac.jp (T.S.); 2 Institute of Nano-Life-Systems, Institutes of Innovation for Future Society, Nagoya University, Furo-cho, Chikusa-ku, Nagoya 464-8603, Japan; 3 Japan Science and Technology Agency (JST), Precursory Research for Embryonic Science and Technology (PRESTO), Kawaguchi, Saitama 332-0012, Japan; kazu-n@g.ecc.u-tokyo.ac.jp (K.N.); takahashi-t@g.ecc.u-tokyo.ac.jp (T.T.); 4 Department of Applied Chemistry, Graduate School of Engineering, The University of Tokyo, Tokyo 113-8656, Japan; yanagida@g.ecc.u-tokyo.ac.jp; 5 Institute for Materials Chemistry and Engineering, Kyushu University, Kasuga, Fukuoka 816-8580, Japan; 6 Institute of Quantum Life Science, National Institutes for Quantum and Radiological Science and Technology, Anagawa 4-9-1, Inage-ku, Chiba 263-8555, Japan

**Keywords:** In_2_O_3_ nanolines, robust, field effect transistor, biosensor platform

## Abstract

Field-effect transistors (FETs) are attractive biosensor platforms for rapid and accurate detection of various analytes through surface immobilization of specific bio-receptors. Since it is difficult to maintain the electrical stability of semiconductors of sensing channel under physiological conditions for long periods, passivation by a stable metal oxide dielectric layer, such as Al_2_O_3_ or HfO_2_, is currently used as a common method to prevent damage. However, protecting the sensing channel by passivation has the disadvantage that the distance between the target and the conductive channel increases, and the sensing signal will be degraded by Debye shielding. Even though many efforts use semiconductor materials directly as channels for biosensors, the electrical stability of semiconductors in the physiological environments has rarely been studied. In this work, an In_2_O_3_ nanolines FET device with high robustness in artificial physiological solution of phosphate buffered saline (PBS) was fabricated and used as a platform for biosensors without employing passivation on the sensing channel. The FET device demonstrated reproducibility with an average threshold voltage (V_TH_) of 5.235 V and a standard deviation (SD) of 0.382 V. We tested the robustness of the In_2_O_3_ nanolines FET device in PBS solution and found that the device had a long-term electrical stability in PBS with more than 9 days’ exposure. Finally, we demonstrated its applicability as a biosensor platform by testing the biosensing performance towards miR-21 targets after immobilizing the phosphonic acid terminated DNA probes. Since the surface immobilization of multiple bioreceptors is feasible, we demonstrate that the robust In_2_O_3_ FET device can be an excellent biosensor platform for biosensors.

## 1. Introduction

Biosensors use biological receptors as their recognition elements in a signal transducing platform that generates a measurable signal in response to changes in the concentration of a given biomolecule [1,2,3,4,5]. Field-effect transistors (FETs) are attractive biosensor platforms for rapid and accurate detection of various analytes through surface immobilization of specific bio-receptors [6,7,8,9]. However, most semiconductor materials of sensing channels cannot be maintained under physiological conditions for long periods of time [10,11]. To solve this problem, surface passivation using a stable metal oxide-based dielectric layer, such as Al_2_O_3_ or HfO_2_, is commonly employed [12,13]. One representative report by Lieber’s group showed that electronic devices based on Si, Ge/Si and InAs nanowires have long-term stability of at least 100 days when passivating the nanowires with ultra-thin (10 nm) Al_2_O_3_ shells [14]. However, protecting the sensing channel under physiological conditions by passivation is disadvantageous because it increases the distance between the charged target and the conductive channel. This can lead to a decrease in sensing sensitivity due to the effect of the Debye shielding length [15,16]. Therefore, robust semiconductor materials for sensing channels that can tolerate long-term use in PBS solution are highly desired.

Semiconductor metal oxides are one of the key compounds for the development of sensitive materials for sensors due to a unique set of properties, especially their electrical conductivity and high reactivity of the surface in the interaction with the molecules [17,18]. Our group have also taken an interest and put a lot of effort into improving the electrical stability of semiconductor metal oxides under air and liquid conditions. Yan et al. [19,20] demonstrated an air/Zn vapor annealing method to obtain a highly conductive Al-doped ZnO (AZO) nanofilm that can maintain its electrical conductivity in air for a long time and even in liquids of different pH. However, the long-term stability in liquids is only increased by 1 to 2 h which is insufficient to meet the needs of biosensor devices, because the immobilization process is carried out in a liquid for more than 2 h, and the fabricated device has to be kept in the physiological solution to maintain the stability of the bioreceptors. In addition, maintaining electrical stability in physiological environments is more challenging due to the presence of various ions and biomolecules. Therefore, the development of stable FET-based metal oxide biosensors requires that more stable materials are available for use as the sensing channels. Although many metal oxide semiconductor-based nanostructures have been reported for use on biosensors, the details of the electrical stability of these oxide semiconductors under physiological conditions have rarely been studied [21,22,23].

In this work, an In_2_O_3_ nanolines FET device with high robustness in artificial physiological solution of PBS was fabricated and used as a platform for biosensors without passivation on the sensing channel. The fabricated FET device demonstrated good reproducibility with a small standard deviation of threshold voltage. We tested the robustness of the In_2_O_3_ nanolines FET device in PBS solution and found that the device had a long-term electrical stability in PBS with more than 9 days’ exposure. Finally, we demonstrated its applicability as a biosensor platform by testing the biosensing performance towards cancer-related biomarker of miR-21 after immobilizing the phosphonic acid terminated DNA probe on the In_2_O_3_ nanolines FET platform.

## 2. Experimental

### 2.1. Formation of In_2_O_3_ Nanolines

**Figure 1:**
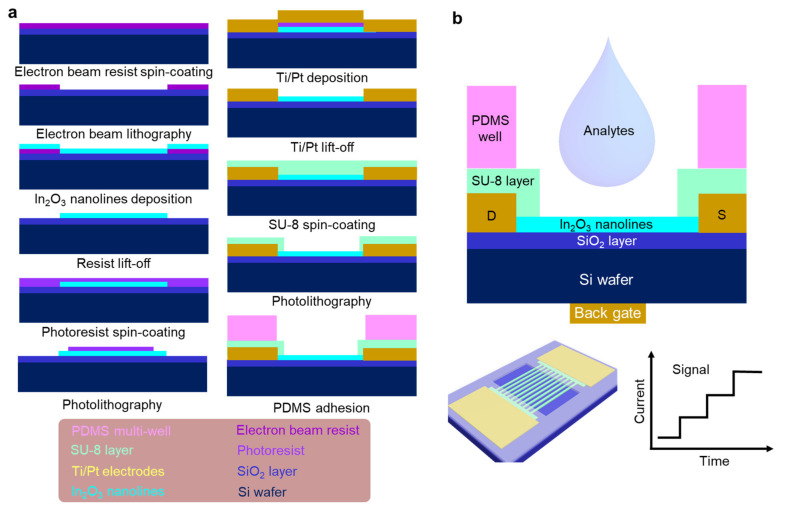
(**a**) Flowchart of the fabrication process of In_2_O_3_ nanolines on Si/SiO_2_ substrate. The materials used in the fabrication are colored-coded in the key. (**b**) Schematic drawings of a cross section and a top view. The signal curve (response) plot of current vs. time when changing the analytes.

The fabrication process of the device was shown in the schematic of Figure 1a. In details, the top surface of a Si wafer (2 × 2 cm) was covered with an SiO_2_ layer (100 nm thick) to obtain the Si/SiO_2_ substrate on which In_2_O_3_ nanolines were deposited. Radio frequency (RF) sputtering and electron beam lithography (EBL) were employed as described in the literature [24,25,26]. In brief, the Si/SiO_2_ substrate was first treated using UV ozone cleaner for 5 min to remove organic contaminates. Then ZEP-50A electron beam resist was deposited on the substrate by a spin coating process (3000 rpm for 200 s). EBL was performed to area-selectively write the In_2_O_3_ line pattern on the substrate using a 30 kV electron beam. After the pattern area was developed using o-xylene developer (2 min), the patterned substrate was transferred to the chamber of an RF sputtering apparatus. Then, 10 nm thick In_2_O_3_ was deposited under the following conditions: power, 30 W; vapor pressure, 1 Pa; O_2_/Ar, 1/5; deposition rate, 2.5 nm/min. The resist was removed in a lift-off process after soaking the substrate in N, N-dimethylformamide for 12 h. The In_2_O_3_ nanolines were cleaned using isopropanol (IPA), followed by drying in air flow. Finally, the substrate with the In_2_O_3_ nanolines was annealed at 400 °C for 1 h in air. ZnO nanolines device was fabricated using the same method. To obtain the conductive device, the thickness of ZnO nanolines was improved to 100 nm. 

### 2.2. Fabrication of In_2_O_3_ Sensing Device

First, the hexamethyldisilazane tackifier was spin coated (3000 rpm for 8 s) on the annealed substrate with the In_2_O_3_ lines, followed by 60 s baking at 105 °C. Then the substrate was spin coated with AZ 5200-E photoresist (1000 rpm for 120 s), followed by 120 s baking at 105 °C. The electrode pattern was exposed using photolithography equipment (Model DDB-700). After developing the pattern, Ti/Pt electrodes were deposited on the device by DC sputtering. Here, the thicknesses of the Ti and Pt layers were 10 nm and 50 nm, respectively. Next, the resist was removed by a lift-off process after soaking in acetone for 12 h. The In_2_O_3_ nanolines FET device was obtained after washing with IPA and drying in N_2_ flow. The FET device was covered with a SU-8 layer to protect the electrodes when the device was soaked in phosphate buffered saline (PBS) solution. In this step, a 5 µm SU-8 layer was spin coated on the device (3000 rpm for 120 s). After baking at 95 °C for 120 s, the In_2_O_3_ line area was exposed using photolithography equipment. After development, a PDMS well was glued on the sensing device to keep the analytes in the In_2_O_3_ line area [27].

### 2.3. Characterizations

The morphologies of the In_2_O_3_ nanolines and other parts of the device such as the Ti and Pt electrodes were observed using an optical microscope and a field-emission scanning electron microscope (FESEM; Zeiss, Supra 40 VP) at the accelerating voltage of 15 kV. Energy-dispersive X-ray spectroscopy (EDS) elemental mapping was done with an FESEM attachment (JEOL, JSM-7610F).

### 2.4. Surface Modifications on Sensing Device

The chemical and biological agents including (3-Aminopropyl)triethoxysilane (APTES), and phosphonic acid terminated DNA (PO_3_H_2_-DNA) probe were individually or multiply modified on the In_2_O_3_ nanolines device. The sequence of DNA of PO_3_H_2_-DNA probe is PHO-AAAAATCAACATCAGTCTGAAGCTA. Before surface modification, all the devices were cleaned by the oxygen plasma for 1 min under 80 W. APTES modified In_2_O_3_ nanolines were obtained by immersing the device in an ethanol solution containing 1% APTES for one hour. Immobilization of PO_3_H_2_-DNA probe on In_2_O_3_ surface was performed by immersing the device in a PBS solution containing the DNA probe (100 µM) and kept it for 5 h. Co-modification of APTES and DNA probe was also performed by immersing in APTES for 1 h, followed by immersing in DNA probe contained PBS solution for 5 h.

### 2.5. Sensing Measurements

The electrical conductance, transfer curve and sensing signal were measured by two connected semiconductor parameter analyzers (Keithley, Model 2401). The analyte was prepared by dispersing miR-21 target in PBS solution (0.01 X). The analyte was dropped on the sensing device by the pipette. The volume of one droplet was controlled to 3 µL. The strength of electrical sensing response was expressed as (I−I_0_)/I_0_. I_0_ and I are the conductance of sensing channel in diluted PBS solution and analyte containing miR-21 targets, respectively.

## 3. Results and Discussion

Figure 1b shows a cross-sectional view of the In_2_O_3_ nanolines FET sensing device structure we fabricated. During sensing measurement, analytes were directly dropped into the PDMS well on the device using a micropipette. When the concentration of the analyte is changed, the response signal is monitored, as shown by the graph at the bottom right of Figure 1b.

**Figure 2:**
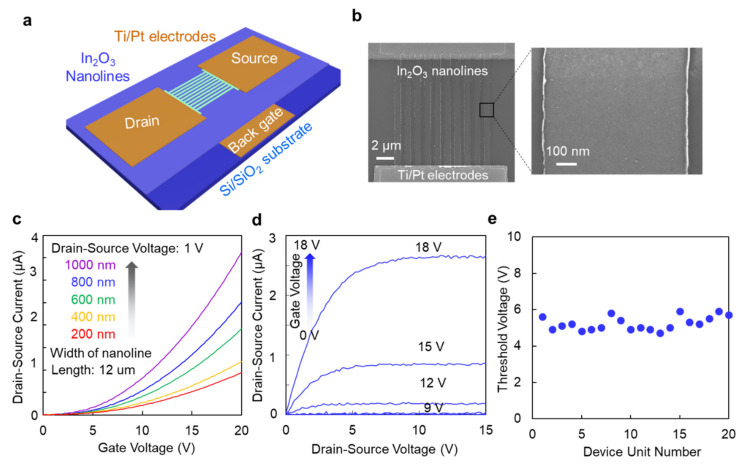
The In_2_O_3_ nanolines FET device. (**a**) Schematic drawing showing the FET device. (**b**) FESEM images of the In_2_O_3_ nanoline area. (**c**) Transfer curve. The FET devices when varying the width of single line were measured. The drain-source voltage was 1 V. (**d**) Output curves. The back-gate voltages were increased from 0 V to 18 V. (**e**) Measured threshold voltages for 20 units of the In_2_O_3_ FET sensing device. The width of device is 800 nm.

We demonstrated performance of the FET device (Figure 2a). As seen from the SEM images (Figure 2b), the two ends of the In_2_O_3_ nanolines were in contact with the Ti/Pt electrodes as the source and drain, respectively, while the Si substrate was in contact with the back gated electrode. To optimize the fabrication process of the device, we measured the transfer curves of In_2_O_3_ nanolines when varying the width of the individual line from 200 nm to 1000 nm. As shown in Figure 2c, the transfer curve shifted to the positive direction when decreasing the width of nanoline, illustrating that nanolines with small widths were easily and completely depleted. The optimized width of each In_2_O_3_ nanoline were set to 800 nm. Furthermore, current modulation when changing the gate voltage was observed from the output curves of the In_2_O_3_ nanolines FET device (Figure 2d). To demonstrate the reproducibility of the FET devices, the threshold voltage (V_TH_) for 20 units of the In_2_O_3_ FET device was measured and plotted in Figure 2e. The V_TH_ was extracted from the linear transfer curve with drain-source voltage (V_DS_) set to 1 V, and the average of 5.235 V and standard deviation of 0.382 V were calculated from the distributed data. Thus, our In_2_O_3_ nanolines FET device demonstrated good reproducibility.

**Figure 3:**
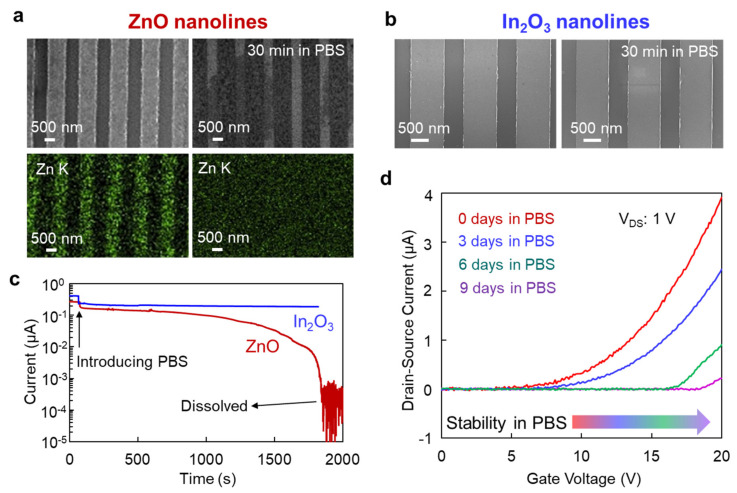
(**a**) FESEM images and EDS elemental mapping images of ZnO lines before and after soaking in PBS solution for 30 min. (**b**) FESEM images of In_2_O_3_ nanolines before and after soaking in PBS solution for 30 min. (**c**) Time dependence of electrical conductance of In_2_O_3_ and ZnO nanolines when soaked in PBS solution. (**d**) Transfer curves of the In_2_O_3_ FET sensing device for different soaking times in PBS. Drain-source voltage was 1 V.

To demonstrate the robustness of our In_2_O_3_ nanolines FET device in PBS solution, we used ZnO nanolines as a comparison, which is an excellent semiconductor metal oxide material for sensing applications. The SEM and EDS mapping images (Figure 3a) showed that the ZnO nanolines were dissolved in PBS. In contrast, there seemed to be little change in the In_2_O_3_ nanolines after immersion under the same conditions (Figure 3b). Next, we measured the time-dependent conductance of ZnO and In_2_O_3_ nanolines in PBS, as shown in Figure 3c. While the ZnO nanolines were broken after PBS immersion for about 1800 s, there was a small change in conductance of the In_2_O_3_ nanolines when they were immersed in PBS. Therefore, we confirmed the robustness of In_2_O_3_ nanolines when immersed in PBS.

Then, we wanted to know how long the In_2_O_3_ FET device could tolerate the PBS solution. We demonstrated the long-term stability of the device by showing time dependent electrical performance when immersed in PBS. The transfer curves of the In_2_O_3_ FET device were measured when soaked in PBS for 3 days, 6 days and 9 days (Figure 3d). When the soaking time was increased, the transfer curve and V_TH_ clearly shifted toward the positive gate voltage. Since In_2_O_3_ might be slowly etched by the ions in the PBS, this degradation in conductive performance was caused by a decrease in the cross-sectional area of the conductive channel. Even so, the In_2_O_3_ FET device maintained its stability for more than 9 days in the PBS solution. Thus, we demonstrated its long-term electrical stability in PBS.

**Figure 4:**
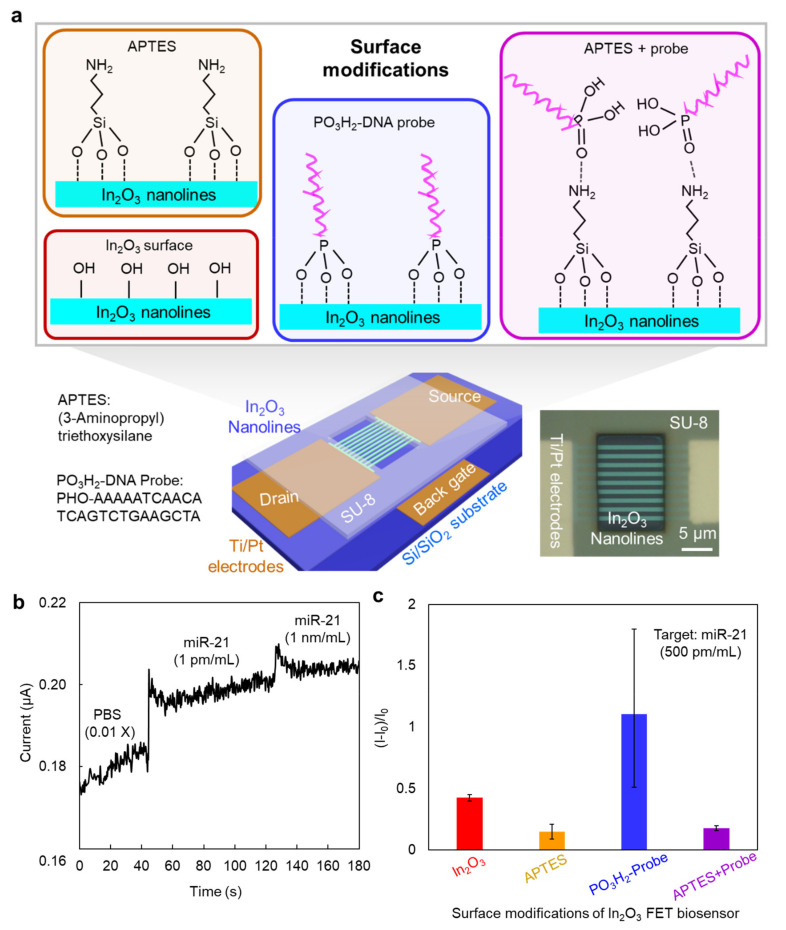
The In_2_O_3_ nanolines FET sensing device. (**a**) Schematics of In_2_O_3_ nanolines FET device and several surface modifications. The information of the APTES and DNA-probe is shown at the lower left. The microscope image of device is in bottom right. (**b**) Representative sensing response curve of DNA probe immobilized In_2_O_3_ sensing device towards miR-21 target. The background solvent is diluted PBS solution (0.01 X). The concentration of miR-21 target is changed. (**c**) Sensing response of the sensors after various surface modifications. The concentration of miR-21 target is 500 pM/mL.

Finally, we tested the applicability of the robust In_2_O_3_ FET device platform to the biosensor by immobilizing the DNA probe towards miR-21 target. As shown in Figure 4a, the FET device was processed into a biosensor device by adding a window formed by a SU-8 protective layer and a PDMS well. Since large distance of the captured targets from the channel decreases the intensity of the sensing signal according to the Debye shielding effect, we chose a phosphonic acid terminated DNA probe in expectation that phosphonic acid group could directly binding to metal oxide surface. [28,29] To demonstrate the effect of DNA probe immobilization on the sensing performance, a common used APTES self-assembled monolayer (SAM) was also modified as a comparison on the In_2_O_3_ sensor channel by a simple immersion process [30]. The details of experiments can be found in experimental section. As shown in Figure 4b, a typical current response signal was observed when the miR-21 analyte (1 pM/mL in 0.01 X PBS solution) was pipetted on the window of In_2_O_3_ nanolines sensor device. To evaluate the detection sensitivity of the devices with various surface modifications, the normalized response of (I−I_0_)/I_0_ was calculated and shown in Figure 4c. The phosphonic acid terminated DNA probe immobilized immunosensors exhibited the stronger sensing response compared to the pristine In_2_O_3_ nanolines sensors, indicating that DNA probes were immobilized on the In_2_O_3_ nanolines and could capture the miR-21 targets and change the conductance of In_2_O_3_ nanolines channel. 

In addition, the sensing response was also higher than the APTES modified sensor, illustrating a better capturing efficiency than the APTES SAM layer towards miR-21. However, a large distribution of sensing response for the DNA probe modified sensors was observed compared to In_2_O_3_ and APTES modified sensors. This might be due to the random binding orientation of DNA probes and the In_2_O_3_ surface. At last, APTES and DNA probe were successively modified on the In_2_O_3_ sensor device to confirm the direct immobilization of DNA probes, as APTES would block the direct binding of phosphonic acid group in DNA probes and In_2_O_3_ channel. We found that the response signal of the sensors was similar in intensity and distribution to the APTES-modified sensors, but weaker than the DNA probe immobilized sensors. This might be because APTES kept the DNA probes away from the In_2_O_3_ nanolines surface, reducing the effect of DNA probe on the sensing response. In other words, the phosphonic acid terminated DNA probe could be directly immobilized on the surface of In_2_O_3_ nanolines sensor platform and effectively captured the miR-21 targets. Since the surface immobilization of multiple bio-probes is feasible, our proposed In_2_O_3_ FET sensing device should be a robust biosensor platform for biosensors.

## 4. Conclusions

In this work, an In_2_O_3_ electrically modulated FET sensing device with high robustness in PBS solution was fabricated and can be used as a platform for biosensors. The fabricated FET device demonstrated reproducibility with an average V_TH_ of 5.235 V and a SD of 0.382 V. We found it retained high robustness when kept in PBS solution for more than 9 days. We demonstrated the FET device had good performance as a biosensor platform by showing its sensing response towards miR-21 after directly immobilized the phosphonic acid terminated DNA probes. Since the surface immobilization of multiple bio-probes is feasible, we demonstrate that our proposed robust In_2_O_3_ nanolines FET device can be an excellent biosensor platform.
